# Hybrid control of the Neimark-Sacker bifurcation in a delayed Nicholson’s blowflies equation

**DOI:** 10.1186/s13662-015-0640-2

**Published:** 2015-10-06

**Authors:** Yuanyuan Wang, Lisha Wang

**Affiliations:** College of Science, China University of Petroleum (East China), Qingdao, 266580 P.R. China; School of Science, Qingdao Technological University, Qingdao, 266033 P.R. China

**Keywords:** hybrid control, nonstandard finite-difference scheme, Neimark-Sacker bifurcation, Nicholson’s blowflies equation, delay

## Abstract

In this article, for delayed Nicholson’s blowflies equation, we propose a hybrid control nonstandard finite-difference (NSFD) scheme in which state feedback and parameter perturbation are used to control the Neimark-Sacker bifurcation. Firstly, the local stability of the positive equilibria for hybrid control delay differential equation is discussed according to Hopf bifurcation theory. Then, for any step-size, a hybrid control numerical algorithm is introduced to generate the Neimark-Sacker bifurcation at a desired point. Finally, numerical simulation results confirm that the control strategy is efficient in controlling the Neimark-Sacker bifurcation. At the same time, the results show that the NSFD control scheme is better than the Euler control method.

## Introduction

The delay differential equation (DDE)

1.1 $$ \dot{x}(t)=ax(t-\tau)e^{-bx(t-\tau)}-cx(t), $$

which is one of the important ecological systems, describes the dynamics of Nicholson’s blowflies equation. Here $x(t)$ is the size of the population at time *t*, a is the maximum per capita daily egg production rate, $1/b$ is the size at which the population reproduces at the maximum rate, *c* is the per capita daily adult death rate, and *τ* is the generation time, the positive equilibrium $x_{\ast}=(1/b)\ln(a/c)$. Equation (1.1) has been extensively studied in the literature. The majority of the results on (1.1) deal with the global attractiveness of the positive equilibrium and oscillatory behaviors of solutions [1, 2].

For experimental or computational purposes, it is common to discretize the continuous-time system corresponding to (1.1). It is desired that the discrete-time model is ‘dynamically consistent’ with the continuous-time model. The aim of bifurcation control is to delay (advance) the onset of an inherent bifurcation, change the parameter value of an existing bifurcation point, stabilize a bifurcated solution or branch, *etc.* [3–8]. In [9–11], the hybrid control strategy is used to control the bifurcation.

We consider the delay differential equation

$$ \dot{u}=f \bigl(u(t),u(t-1) \bigr), \quad t\geq0; \qquad u(t)=\eta(t),\quad -1 \leq t\leq0. $$

The first-order derivative is approximated by the modified forward Euler expression

$$ \frac{du(t)}{dt}\longrightarrow\frac{u_{k+1}-u_{k}}{\phi}, $$

with the ‘denominator function’ *ϕ* such that

$$ \phi(h)=h+O \bigl(h^{2} \bigr), $$

where $h=1/m$ stands for step-size and $u_{k}$ denotes the approximate value to $u(kh)$, so we get the method as follows:

$$ u_{k+1}-u_{k}=\phi(h)f(u_{k},u_{k-m}). $$

A class of numerical methods, named nonstandard finite difference (NSFD) methods by Mickens, perform well in preserving the properties of the corresponding continuous system [12]. Some NSFD methods have received considerable attention due to the improvement in their efficient computation. NSFD scheme [13–17] tries to preserve the significant properties of their continuous analogues and, consequently, gives reliable numerical results.

In [18], for sufficiently small step-size, the discrete model undergoes a Hopf bifurcation of the same type with the original model by using the Euler forward method. In this paper, we construct a hybrid control nonstandard finite-difference scheme in which state feedback and parameter perturbation are used to control the Neimark-Sacker bifurcations. The results show that the dynamic behavior of a controlled system can be changed by choosing appropriate control parameters. For any step-size, we obtain the consistent dynamical results of the corresponding continuous-time model. To the best of our knowledge, to this day, by the hybrid control NSFD method there are few results dealing with numerical controlled dynamics for DDEs.

The rest of this paper is organized as follows. In Section 2, we summarize the existence and stability of equilibria for the original system (1.1) (Ref. [19]). In Section 3, we analyze the distribution of the characteristic equation associated with a hybrid control delay differential equation with Nicholson’s blowflies equation, and we obtain local stability of the equilibria and existence of the Hopf bifurcation. In Section 4, a hybrid control numerical algorithm is introduced to generate the Neimark-Sacker bifurcation at a desired bifurcation point. In Section 5, the direction and stability of bifurcating periodic solutions from the Neimark-Sacker bifurcation of a controlled delay equation are determined by using the theories of discrete systems. In Section 6, some computer simulations are performed to illustrate the theoretical results. The results show that the NSFD control scheme is better than the Euler control method.

## Existence and stability of equilibria

In the original delay differential equation model (1.1), the time delay *τ* acts as a bifurcation parameter. As the delay *τ* passes through some critical value $\tau_{k}$, a couple of complex conjugating eigenvalues of the system pass the imaginary axis at some pure imaginary points, and stable periodic Hopf bifurcating solutions occur. Then, when *τ* passes $\tau_{k}$, the real parts of these eigenvalues pass to the positive real axis causing the Hopf bifurcating solution to be unstable. We summarize these features of the solution via the existence and stability of a positive equilibrium following the works in [19], Theorem 2.3.

In summary:

- If $c< a< ce^{2}$, then $x=x_{\ast}$ is asymptotically stable.
- If $a>ce^{2}$ ($bx_{\ast}>2$), then $x=x_{\ast}$ is asymptotically stable for $\tau\in[0,\tau_{0})$ and unstable for $\tau>\tau_{0}$. Equation (1.1) undergoes a Hopf bifurcation at $x=x_{\ast}$ when $\tau=\tau_{k}$ for $k=0, 1, 2,\ldots$ .

## Hopf bifurcation in hybrid control DDE

Let $u(t)=x(\tau t)$. Then Eq. (1.1) can be rewritten as

3.1 $$ \dot{u}(t)=a\tau u(t-1)e^{-bu(t-1)}-c \tau u(t). $$

One can see that if $u_{\ast}$ is a positive fixed point to Eq. (3.1), then $u_{\ast}$ satisfies

3.2 $$ c=ae^{-bu_{\ast}}, $$

here $u_{\ast}=x_{\ast}$. Apply both parameter perturbation and state feedback to system (3.1) as follows:

3.3 $$ \dot{u}(t)=\alpha \bigl[a\tau u(t-1)e^{-bu(t-1)}-c\tau u(t) \bigr]+(1-\alpha)\tau \bigl(u(t-1)-u_{\ast} \bigr), \quad 0< \alpha\leq1. $$

Set $z(t)=u(t)-u_{\ast}$. Equation (3.3) becomes

3.4 $$ \dot{z}(t)=\alpha \bigl[a\tau \bigl(z(t-1)+u_{\ast} \bigr)e^{-b(z(t-1)+u_{\ast})}-c\tau \bigl(z(t)+u_{\ast} \bigr) \bigr]+(1-\alpha) \tau z(t-1). $$

The linearization of Eq. (3.4) at $z=0$ is

3.5 $$ \dot{z}(t)=\alpha c\tau \bigl[(1-bu_{\ast})z(t-1)-z(t) \bigr]+(1-\alpha)\tau z(t-1), $$

whose characteristic equation is

3.6 $$ \lambda=\alpha c\tau \bigl[(1-bu_{\ast})e^{-\lambda}-1 \bigr]+(1-\alpha)\tau e^{-\lambda}. $$

When $bu_{\ast}>\frac{1-\alpha}{\alpha c}$, $\lambda<0$. For $\omega \neq0$, i*ω* is a root of Eq. (3.6) if and only if

$$ \mathrm{i}\omega=\alpha c\tau \bigl[(1-bu_{\ast})e^{-\mathrm{i}\omega }-1 \bigr]+(1-\alpha)\tau e^{-\mathrm{i}\omega}. $$

Separating the real and imaginary parts, we obtain

3.7 $$ \textstyle\begin{cases} [\alpha c\tau(1-bu_{\ast})+(1-\alpha)\tau]\cos\omega=\alpha c\tau,\\ {[}\alpha c\tau(1-bu_{\ast})+(1-\alpha)\tau]\sin\omega=-\omega, \end{cases} $$

which leads to

$$ \omega^{2}+(\alpha c\tau)^{2}= \bigl[\alpha c \tau(1-bu_{\ast})+(1-\alpha)\tau \bigr]^{2}, $$

that is,

3.8 $$ \omega=\pm\tau\sqrt{ \bigl[(1-\alpha)-\alpha cbu_{\ast} \bigr] \bigl[(1- \alpha)-\alpha c(2-bu_{\ast}) \bigr]}. $$

This is possible if and only if $bu_{\ast}>2+\frac{1-\alpha}{\alpha c}$.

For $bu_{\ast}>2+\frac{1-\alpha}{\alpha c}$, let

$$ \tau_{k}=\arcsin \biggl[-\frac{w}{\alpha c\tau(1-bu_{\ast})+(1-\alpha)\tau } \biggr]+2k\pi,\quad k=0,1,2,\ldots. $$

Set

$$ \omega_{0}=\tau\sqrt{ \bigl[(1-\alpha)-\alpha cbu_{\ast} \bigr] \bigl[(1-\alpha)-\alpha c(2-bu_{\ast}) \bigr]}. $$

Let $\lambda_{k}=\alpha_{k}(\tau)+\mathrm{i}\omega_{k}(\tau)$ denote a root of Eq. (3.6) near $\tau=\tau_{k}$ such that $\alpha_{k}(\tau _{k})=0$, $\omega_{k}(\tau_{k})=\omega_{0}$. We have the following result.

### Lemma 1

$\alpha_{k}'(\tau_{k})>0$.

### Proof

Differentiating both sides of Eq. (3.6) with respect to *τ*, we obtain

$$ \frac{d\lambda}{d\tau}=\frac{[\alpha c(1-bu_{\ast})+(1-\alpha )]e^{-\lambda}-\alpha c}{1+\tau[\alpha c(1-bu_{\ast})+(1-\alpha )]e^{-\lambda}}. $$

Therefore

$$ \frac{d\lambda}{d\tau}\bigg|_{\tau=\tau_{k}}=\frac{[\alpha c(1-bu_{\ast })+(1-\alpha)]\cos\omega_{0}-\alpha c- \mathrm{i}[\alpha c(1-bu_{\ast })+(1-\alpha)]\sin\omega_{0} }{1+\tau_{k}[\alpha c(1-bu_{\ast })+(1-\alpha)]\cos\omega_{0}-\mathrm{i}\tau_{k}[\alpha c(1-bu_{\ast })+(1-\alpha)]\sin\omega_{0} }. $$

This implies that

$$\begin{aligned} \alpha_{k}'( \tau_{k})&=\frac{[\alpha c(1-bu_{\ast})+(1-\alpha)]\cos \omega_{0}+\tau_{k}[\alpha c(1-bu_{\ast})+(1-\alpha)]^{2}}{\Delta} \\ & =\frac{\alpha c+\tau_{k}[\alpha c(1-bu_{\ast })+(1-\alpha)]^{2}}{\Delta}, \end{aligned}$$

where $\Delta=[1+\tau_{k}[\alpha c(1-bu_{\ast})+(1-\alpha)]\cos\omega _{0}]^{2}+[\tau_{k}[\alpha c(1-bu_{\ast})+(1-\alpha)]\sin\omega _{0}]^{2}$, completing the proof. □

### Theorem 1

*For system* (3.3), *the following statements are true*:

- *If*$\frac{1-\alpha}{\alpha c}< bu_{\ast}<2+\frac{1-\alpha}{\alpha c}$, *then*$u=u_{\ast}$*is asymptotically stable*.
- *If*$bu_{\ast}>2+\frac{1-\alpha}{\alpha c}$ (*when*$\alpha=1$, $bx_{\ast}>2$), *then*$u=u_{\ast}$*is asymptotically stable for*$\tau\in[0,\tau_{0})$*and unstable for*$\tau>\tau_{0}$. *Equation* (3.3) *undergoes a Hopf bifurcation at*$u=u_{\ast}$*when*$\tau=\tau_{k}$*for*$k=0, 1, 2,\ldots$ .

## Stabilization of NSFD hybrid control system

In this section, we mainly discuss the stability and bifurcation of the numerical discrete hybrid control system. We implement the hybrid control strategy [9–11]. Set $v(t)=u(t)-u_{\ast}$. Equation (3.3) becomes

4.1 $$ \dot{v}(t)=\alpha \bigl[a\tau \bigl(v(t-1)+u_{\ast} \bigr)e^{-b(v(t-1)+u_{\ast})}-c\tau \bigl(v(t)+u_{\ast} \bigr) \bigr]+(1-\alpha) \tau v(t-1),\quad 0< \alpha\leq1. $$

When $\alpha=1$, Eq. (4.1) is the uncontrolled system. The differential equation

$$ \frac{dv}{dt}=-\alpha c\tau v(t) $$

has the general solution $v(t)=\bar{C}e^{-\alpha c\tau t}$. We consider step-size of the form $h=1/m$, where $m\in Z_{+}$. The solution can be written as

$$ \frac{v(t+h)-v(t)}{\frac{1-e^{-\alpha c \tau h}}{\alpha c\tau }}=-\alpha c\tau v(t). $$

This is an exact finite difference numerical method:

$$\begin{aligned} v(t+h)-v(t)&=\bar{C}e^{-\alpha c\tau(t+h)}- \bar{C}e^{-\alpha c\tau t} \\ & =\bar{C}e^{-\alpha c\tau t} \bigl(e^{-\alpha c\tau h}-1 \bigr) \\ & =-\alpha c\tau v(t)\frac {1-e^{-\alpha c\tau h}}{\alpha c\tau}. \end{aligned}$$

Employ the NSFD scheme [13, 14, 17] to Eq. (4.1) and choose the ‘denominator function’ *ψ* as

4.2 $$ \psi(h)=\frac{1-e^{-\alpha c\tau h}}{\alpha c\tau}. $$

It yields the difference equation

4.3 $$\begin{aligned} v_{n+1}={}&e^{-\alpha c\tau h}v_{n}+ \bigl(e^{-\alpha c\tau h}-1 \bigr)u_{\ast }+ \bigl(1-e^{-\alpha c\tau h} \bigr) (v_{n-m}+u_{\ast})e^{-bv_{n-m}} \\ &{}+\frac {(1-e^{-\alpha c\tau h})(1-\alpha)}{\alpha c}v_{n-m}. \end{aligned}$$

Introducing a new variable $V_{n}=(v_{n},v_{n-1},\ldots,v_{n-m})^{T}$, we can rewrite (4.3) as

4.4 $$ V_{n+1}=\bar{F}(V_{n},\tau), $$

where $\bar{F}=(\bar{F}_{0},\bar{F}_{1},\ldots,\bar{F}_{m})^{T}$, and

4.5 $$ \bar{F}_{k}= \textstyle\begin{cases} e^{-\alpha c\tau h}v_{n-k}+(e^{-\alpha c\tau h}-1)u_{\ast} +(1-e^{-\alpha c\tau h})(v_{n-m-k}+u_{\ast})e^{-bv_{n-m-k}}\\ \quad{}+\frac{(1-e^{-\alpha c\tau h})(1-\alpha)}{\alpha c}v_{n-m-k},\quad k=0,\\ v_{n-k+1}, \quad 1\leq k \leq m. \end{cases} $$

Clearly the linear part of map (4.4) is

4.6 $$ V_{n+1}=\tilde{A}V_{n}. $$

Here

4.7 $$ \tilde{A}= \begin{bmatrix} e^{-\alpha c\tau h}&0&\cdots&0&0&(1-e^{-\alpha c\tau h})(1-bu_{\ast }+\frac{1-\alpha}{\alpha c}) \\ 1&0&\cdots&0&0&0\\ 0&1&\cdots&0&0&0\\ \dots & \dots & \dots &\dots &\dots & \dots\\ 0&0&\cdots&1&0&0\\ 0&0&\cdots&0&1&0 \end{bmatrix}. $$

The characteristic equation of *Ã* is

4.8 $$ \lambda^{m+1}-e^{-\alpha c\tau h}\lambda^{m}- \bigl(1-e^{-\alpha c\tau h} \bigr) \biggl(1-bu_{\ast}+\frac{1-\alpha}{\alpha c} \biggr)=0. $$

### Lemma 2

*If*$bu_{\ast}>\frac{1-\alpha}{\alpha c}$, *then all roots of Eq*. (4.8) *have modulus less than one for sufficiently small*$\tau>0$.

### Proof

For $\tau=0$, Eq. (4.8) becomes

$$ \lambda^{m+1}-\lambda^{m}=0. $$

The equation has an *m*-fold root $\lambda=0$ and a simple root $\lambda=1$.

Consider the root $\lambda(\tau)$ such that $|\lambda(0)|= 1$. This root is a $C^{1}$ function of *τ*. For Eq. (4.8), we have

$$\begin{aligned}& \frac{d|\lambda|^{2}}{d\tau}=\lambda\frac{d\overline{\lambda}}{d\tau }+\overline{\lambda} \frac{d\lambda}{d\tau}, \\& \frac{d|\lambda|^{2}}{d\tau}\bigg|_{\lambda=1,\tau=0}=2h(1-\alpha-bu_{\ast }\alpha c). \end{aligned}$$

Consequently, if $1-\alpha-bu_{\ast}\alpha c<0$, then all roots of Eq. (4.8) lie in $|\lambda|<1$ for sufficiently small $\tau>0$. □

### Lemma 3

*For any step*-*sizeh*, *if*$\frac{1-\alpha}{\alpha c}< bu_{\ast}<2+\frac{1-\alpha}{\alpha c}$, *then Eq*. (4.8) *has no root with modulus one for all*$\tau>0$.

### Proof

A Neimark-Sacker bifurcation occurs when two roots of the characteristic equation (4.8) cross the unit circle. We have to find values of *τ* such that there exist roots on the unit circle. The roots on the unit circle are given by $e^{\mathrm{i}\omega}$, $\omega\in (-\pi,\pi]$. Since we are dealing with a real polynomial, complex roots occur in complex conjugate pairs and we have only to look for $\omega\in(0,\pi]$. For $\omega\in(0,\pi]$, $e^{\mathrm{i}\omega}$ is a root of (4.8) if and only if

4.9 $$ e^{\mathrm{i}\omega}-e^{-\alpha c\tau h}- \bigl(1-e^{-\alpha c\tau h} \bigr) \biggl(1-bu_{\ast}+\frac{1-\alpha}{\alpha c} \biggr)e^{-\mathrm{i}m\omega}=0. $$

Hence

4.10 $$ \textstyle\begin{cases} \cos\omega-e^{-\alpha c\tau h}-(1-e^{-\alpha c\tau h})(1-bu_{\ast }+\frac{1-\alpha}{\alpha c})\cos m\omega=0,\\ \sin\omega+(1-e^{-\alpha c\tau h})(1-bu_{\ast}+\frac{1-\alpha}{\alpha c})\sin m\omega=0. \end{cases} $$

We obtain

4.11 $$ \cos\omega=1+\frac{(1-e^{-\alpha c\tau h})^{2}(bu_{\ast}-\frac{1-\alpha}{\alpha c})(2+\frac{1-\alpha}{\alpha c}-bu_{\ast})}{2e^{-\alpha c\tau h}}. $$

If $\frac{1-\alpha}{\alpha c}< bu_{\ast}<2+\frac{1-\alpha}{\alpha c}$, then $\cos\omega>1$, which yields a contradiction. So Eq. (4.8) has no root with modulus one for all $\tau>0$. □

For $bu_{\ast}>2+\frac{1-\alpha}{\alpha c}$, for any step-size *h*, $|\cos\omega|<1$ and $\tau>0$ is positive real, from (4.11) we know that

4.12 $$\begin{aligned} &\omega_{k}=\arccos \biggl(1+\frac{(1-e^{-\alpha c\tau h})^{2}(bu_{\ast}-\frac{1-\alpha}{\alpha c})(2+\frac{1-\alpha}{\alpha c}-bu_{\ast})}{2e^{-\alpha c\tau h}} \biggr)+2k \pi, \\ &\quad k=0,1,2,\ldots, \biggl[\frac{m-1}{2} \biggr], \end{aligned}$$

where $[\cdot]$ denotes the greatest integer function. It is clear that there exists a sequence of the time delay parameters $\tau_{k}$ satisfying Eq. (4.10) according to $\omega=\omega_{k}$.

### Lemma 4

*For any step*-*sizeh*, *if*$bu_{\ast}>2+\frac{1-\alpha }{\alpha c}$, *let*$\lambda_{k}(\tau)=r_{k}(\tau)e^{\mathrm{i}\omega _{k}(\tau)}$*be a root of Eq*. (4.8) *near*$\tau=\tau_{k}$*satisfying*$r_{k}(\tau_{k})=1$*and*$\omega_{k}(\tau_{k})=\omega_{k}$, *then*$\frac{dr_{k}^{2}(\tau)}{d\tau}|_{\tau=\tau_{k}, \omega=\omega_{k}}>0$.

### Proof

From Eq. (4.8), we obtain

$$\begin{aligned}& \lambda^{m}=\frac{(1-e^{-\alpha c\tau h})(1+\frac{1-\alpha}{\alpha c}-bu_{\ast})}{\lambda-e^{-\alpha c\tau h}}, \\& \frac{dr_{k}^{2}(\tau)}{d\tau}\bigg|_{\tau=\tau_{k}, \omega=\omega _{k}}=2\Re \biggl( \overline{\lambda} \frac{d\lambda}{d\tau} \biggr)\bigg|_{\tau=\tau_{k}, \omega=\omega_{k}} \\& \hphantom{\frac{dr_{k}^{2}(\tau)}{d\tau}\bigg|_{\tau=\tau_{k}, \omega=\omega _{k}}}=\frac{2\alpha che^{-\alpha c\tau h}(m+1+me^{-\alpha c\tau h})(1-\cos \omega)}{(1-e^{-\alpha c\tau h})[((m+1)\cos\omega-me^{-\alpha c\tau h})^{2}+((m+1)\sin\omega)^{2}]}\bigg|_{\tau=\tau_{k}, \omega=\omega_{k}}\\& \hphantom{\frac{dr_{k}^{2}(\tau)}{d\tau}\bigg|_{\tau=\tau_{k}, \omega=\omega _{k}}}>0. \end{aligned}$$

This completes the proof. □

### Theorem 2

*For system* (4.3), *the following statements are true*:

1. *If*$\frac{1-\alpha}{\alpha c}< bu_{\ast}<2+\frac{1-\alpha}{\alpha c}$, *then*$u=u_{\ast}$*is asymptotically stable for any*$\tau>0$;
2. *If*$bu_{\ast}>2+\frac{1-\alpha}{\alpha c}$, *then*$u=u_{\ast}$*is asymptotically stable for*$\tau\in(0,\tau_{0})$*and unstable for*$\tau>\tau_{0}$. *Equation* (4.3) *undergoes a Neimark*-*Sacker bifurcation at*$u=u_{\ast}$*when*$\tau=\tau_{k}$*for*$k=0,1,2,\ldots,[\frac{m-1}{2}]$.

### Proof

(1) If $\frac{1-\alpha}{\alpha c}< bu_{\ast}<2+\frac{1-\alpha}{\alpha c}$, from Lemmas 2 and 3 we know that Eq. (4.8) has no root with modulus one for all $\tau>0$. Applying Corollary 2.4 in [20], all roots of Eq. (4.8) have modulus less than one for all $\tau>0$. The conclusion follows.

(2) If $bu_{\ast}>2+\frac{1-\alpha}{\alpha c}$, applying Lemmas 2 and 4, we know that all roots of Eq. (4.8) have modulus less than one when $\tau\in(0,\tau_{0})$, and Eq. (4.8) has at least a couple of roots with modulus greater than one when $\tau>\tau_{0}$. The conclusion follows. □

### Remark 1

According to the conclusions of Lemmas 2-4 and Theorem 2, for any step-size, due to $bu_{\ast}>2+\frac{1-\alpha}{\alpha c}$, we can delay the onset of a Neimark-Sacker bifurcation by choosing different *α* ($0<\alpha<1$).

## Direction and stability of the Neimark-Sacker bifurcation in discrete control model

In this section, we discuss direction and stability of the Neimark-Sacker bifurcation in a discrete control system. In Section 4, we obtained conditions for the Neimark-Sacker bifurcation to occur when $\tau=\tau_{k}$ for $k=0,1,2,\ldots,[\frac{m-1}{2}]$. In this section we study the direction of the Neimark-Sacker bifurcation and the stability of the bifurcating periodic solutions when $\tau=\tau_{0}$, using techniques from normal form and center manifold theory [21, 22].

$$\begin{aligned} v_{n+1}={}&e^{-\alpha c\tau h}v_{n}+ \bigl(1-e^{-\alpha c\tau h} \bigr) \biggl(1+\frac {1-\alpha}{\alpha c}-bu_{\ast} \biggr) v_{n-m} \\ &{}+\frac{1}{2} \bigl(1-e^{-\alpha c\tau h} \bigr) \bigl(b^{2}u_{\ast}-2b \bigr)v_{n-m}^{2}+\frac{1}{6} \bigl(1-e^{-\alpha c\tau h} \bigr) \bigl(3b^{2}-b^{3}u_{\ast} \bigr)v_{n-m}^{3}+O \bigl(|x_{n-m}|^{4} \bigr). \end{aligned}$$

So, we can write system (4.3) as

$$ V_{n+1}=\tilde{A}V_{n}+\frac{1}{2}B(V_{n},V_{n})+ \frac {1}{6}C(V_{n},V_{n},V_{n})+O \bigl( \|V_{n} \|^{4} \bigr), $$

where

$$\begin{aligned}& B(V_{n},V_{n})= \bigl(b_{0}(V_{n},V_{n}),0, \ldots,0 \bigr)^{T}, \qquad C(V_{n},V_{n},V_{n})= \bigl(c_{0}(V_{n},V_{n},V_{n}),0, \ldots,0 \bigr)^{T}, \end{aligned}$$

and

5.1 $$ \begin{aligned} &\tilde{a}_{0}=e^{-\alpha c\tau h}, \\ &\tilde{a}_{1}= \bigl(1-e^{-\alpha c\tau h} \bigr) \biggl(1+ \frac{1-\alpha}{\alpha c}-bu_{\ast} \biggr), \\ &b_{0}(\phi,\phi)= \bigl(1-e^{-\alpha c\tau h} \bigr) \bigl(b^{2}u_{\ast}-2b \bigr)\phi _{m}^{2}= \widetilde{b}\cdot\phi_{m}^{2}, \\ & c_{0}(\phi,\phi,\phi)= \bigl(1-e^{-\alpha c\tau h} \bigr) \bigl(3b^{2}-b^{3}u_{\ast } \bigr) \phi_{m}^{3} =\widetilde{c}\cdot\phi_{m}^{3}. \end{aligned} $$

Let $q=q(\tau_{0})\in \mathbb{C}^{m+1}$ be an eigenvector of *Ã* corresponding to $e^{\mathrm{i}\omega_{0}}$, then

$$\tilde{A}q=e^{\mathrm{i}\omega_{0}}q, \qquad \tilde{A}\overline {q}=e^{-\mathrm{i}\omega_{0}} \overline{q}. $$

We also introduce an adjoint eigenvector $q^{\ast}=q^{\ast}(\tau)\in {\mathbb{C}^{m+1}}$ having the properties

$$\tilde{A}^{T}q^{\ast}=e^{-\mathrm{i}\omega_{0}}q^{\ast},\qquad \tilde{A}^{T}\overline{q}^{\ast}=e^{\mathrm{i}\omega_{0}} \overline {q}^{\ast}, $$

and satisfying the normalization $\langle q^{\ast},q\rangle=1$, where $\langle q^{\ast},q\rangle=\sum_{i=0}^{m}\overline{q}_{i}^{\ast}q_{i}$.

### Lemma 5

([23])

*Define a vector*-*valued function*$p: \mathbb{C} \longrightarrow\mathbb{C}^{m+1}$*by*

$$p(\xi)= \bigl(\xi^{m},\xi^{m-1},\ldots,1 \bigr)^{T}. $$

*Ifξis an eigenvalue ofÃ*, *then*$\tilde{A}p(\xi)=\xi p(\xi)$.

In view of Lemma 5, we have

5.2 $$ q=p \bigl(e^{\mathrm{i}w_{0}} \bigr)= \bigl(e^{\mathrm{i}mw_{0}},e^{\mathrm {i}(m-1)w_{0}}, \ldots,e^{\mathrm{i}w_{0}},1 \bigr)^{T}. $$

### Lemma 6

*Suppose*$q^{\ast}=(q_{0}^{\ast},q_{1}^{\ast},\ldots ,q_{m}^{\ast})^{T}$*is the eigenvector of*$\tilde{A}^{T}$*corresponding to the eigenvalue*$e^{-\mathrm{i}w_{0}}$, *and*$\langle q^{\ast},q\rangle=1$. *Then*

5.3 $$ q^{\ast}=\overline{K} \bigl(1,\tilde{a}_{1}e^{\mathrm{i}mw_{0}}, \tilde {a}_{1}e^{\mathrm{i}(m-1)w_{0}},\ldots,\tilde{a}_{1}e^{2\mathrm {i}w_{0}}, \tilde{a}_{1}e^{\mathrm{i}w_{0}} \bigr)^{T}, $$

*where*

5.4 $$ K= \bigl[e^{-\mathrm{i}mw_{0}}+m\tilde{a}_{1}e^{-\mathrm{i}w_{0}} \bigr]^{-1}. $$

### Proof

Assign $q^{\ast}$ satisfies $\tilde{A}^{T}q^{\ast}=\overline{z}q^{\ast}$ with $\overline{z}=e^{-\mathrm{i}w_{0}}$. Then there are

5.5 $$ \textstyle\begin{cases} \tilde{a}_{0}q_{0}^{\ast}+q_{1}^{\ast}=e^{-\mathrm{i}w_{0}}q_{0}^{\ast},\\ q_{k}^{\ast}=e^{-\mathrm{i}w_{0}}q_{k-1}^{\ast},\quad k=2,3,\ldots,m,\\ \tilde{a}_{1}q_{0}^{\ast}=e^{-\mathrm{i}w_{0}}q_{m}^{\ast}. \end{cases} $$

Let $q_{m}^{\ast}=\tilde{a}_{1}e^{\mathrm{i}w_{0}}\overline{K}$, by the normalization $\langle q^{\ast},q\rangle=1$ and direct computation, the lemma follows. □

Let $T_{\mathrm{center}}$ denote a real eigenspace corresponding to $e^{\pm\mathrm{i}w_{0}}$, which is two-dimensional and is spanned by $\{\operatorname{Re}(q),\operatorname{Im}(\bar{q})\}$, and let $T_{\mathrm{stable}}$ be a real eigenspace corresponding to all eigenvalues of $\tilde{A}^{T}$, other than $e^{\pm\mathrm{i}w_{0}}$, which is $(m-1)$-dimensional.

All vectors $x\in\mathbb{R}^{m+1}$ can be decomposed as

$$ x=vq+\bar{v} \bar{q}+y, $$

where $v\in\mathbb{C}$, $vq+\bar{v} \bar{q}\in T_{\mathrm{center}}$, and $y\in T_{\mathrm{stable}}$. The complex variable *v* can be viewed as a new coordinate on $T_{\mathrm{center}}$, so we have

$$\begin{aligned} &v= \bigl\langle q^{_{\ast}},x \bigr\rangle , \\ &y=x- \bigl\langle q^{_{\ast}},x \bigr\rangle q- \bigl\langle \overline{q}^{_{\ast}},x \bigr\rangle \overline{q}. \end{aligned}$$

Let $a(\lambda)$ be characteristic polynomial of *Ã* and $\lambda_{0}=e^{\mathrm{i}w_{0}}$. Following the algorithms in [21] and using a computation process similar to that in [18, 23], we have

$$\begin{aligned} &g_{20}= \bigl\langle q^{\ast},B(q,q) \bigr\rangle , \\ &g_{11}= \bigl\langle q^{\ast},B(q,\overline{q}) \bigr\rangle , \\ &g_{02}= \bigl\langle q^{\ast},B(\overline{q},\overline{q}) \bigr\rangle , \\ &g_{21}= \bigl\langle q^{\ast},B(\overline{q},w_{20}) \bigr\rangle +2 \bigl\langle q^{\ast },B(q,w_{11}) \bigr\rangle + \bigl\langle q^{\ast},C(q,q,\overline{q}) \bigr\rangle , \end{aligned}$$

where

$$\begin{aligned} &w_{20}=\frac{b_{0}(q,q)}{a(\lambda_{0}^{2})}p \bigl( \lambda_{0}^{2} \bigr)-\frac {\langle q^{\ast},B(q,q)\rangle }{\lambda_{0}^{2} -\lambda_{0}}q-\frac{\langle \overline {q}^{\ast},B(q,q)\rangle }{\lambda_{0}^{2} -\overline{\lambda_{0}}}\overline {q}, \\ &w_{11}=\frac{b_{0}(q,\overline{q})}{a(1)}p(1)-\frac{\langle q^{\ast },B(q,\overline{q})\rangle }{1-\lambda_{0}}q-\frac{\langle \overline{q}^{\ast },B(q,\overline{q})\rangle }{1-\overline{\lambda_{0}}} \overline{q}. \end{aligned}$$

So, we can compute an expression for the critical coefficient $c_{1}(\tau_{0})$

5.6 $$ c_{1}(\tau_{0})=\frac{g_{20}g_{11}(1-2\lambda_{0})}{2(\lambda _{0}^{2}-\lambda_{0})}+\frac{|g_{11}|^{2}}{1-\overline{\lambda _{0}}}+ \frac{|g_{02}|^{2}}{2(\lambda_{0}^{2}-\overline{\lambda _{0}})}+\frac{g_{21}}{2}. $$

By (5.1), (5.2) and Lemma 6, we get

5.7 $$ c_{1}(\tau_{0})=\frac{K}{2} \biggl( \frac{\widetilde {b}^{2}}{a(e^{2w_{0}i})}+\frac{2\widetilde{b}^{2}}{a(1)}+\widetilde{c} \biggr). $$

Thus applying the Neimark-Sacker bifurcation theorem [24], the stability of the closed invariant curve can be summarized as follows.

### Theorem 3

*If*$bu_{\ast}>2+\frac{1-\alpha}{\alpha c}$, *then*$u=u_{\ast}$*is asymptotically stable for any*$\tau\in[0,\tau_{0})$*and unstable for*$\tau>\tau_{0}$. *An attracting* (*repelling*) *invariant closed curve exists for*$\tau>\tau_{0}$*if*$\Re[e^{-\mathrm{i}w_{0}}c_{1}(\tau_{0})]<0$ (>0) (*when*$\alpha=1$, *we obtain the results of the uncontrolled system*).

### Remark 2

The parameter *α* could decide the dynamics of system (4.3), *e.g.*, the direction of the bifurcation, the stability and the amplitude of the closed invariant curve.

## Numerical simulations

One of the purposes of this section is to test the results in Sections 3-5 by numerical examples; the second one is to show that the hybrid control NSFD numerical algorithm is better than the Euler control method.

**Figure 1:**
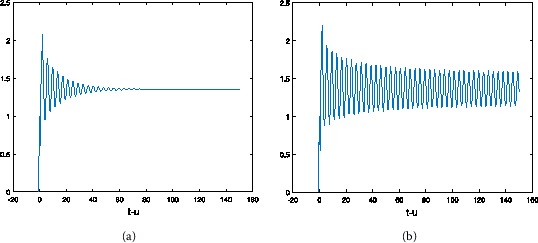
**The numerical solution of Eq. (** **3.3** **) with hybrid control NSFD scheme corresponding to** $\pmb{\alpha=1}$ **,** $\pmb{h=1/2}$ **when (a)** $\pmb{\tau=0.5}$ **, (b)** $\pmb{\tau=0.6}$ **.**

**Figure 2:**
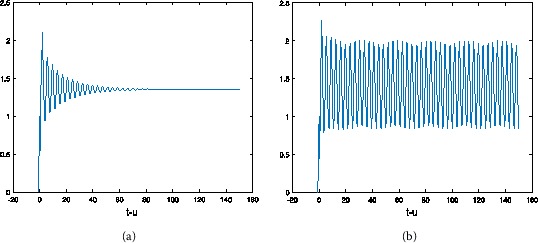
**The numerical solution of Eq. (** **3.3** **) with hybrid control NSFD scheme corresponding to** $\pmb{\alpha=0.9}$ **,** $\pmb{h=1/2}$ **when (a)** $\pmb{\tau=0.6}$ **, (b)** $\pmb{\tau=0.8}$ **.**

**Figure 3:**
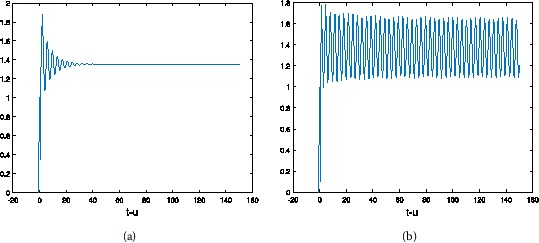
**The numerical solution of Eq. (** **3.3** **) with hybrid control NSFD scheme corresponding to** $\pmb{\alpha=0.6}$ **,** $\pmb{h=1/2}$ **when (a)** $\pmb{\tau=1}$ **, (b)** $\pmb{\tau=1.6}$ **.**

**Figure 4:**
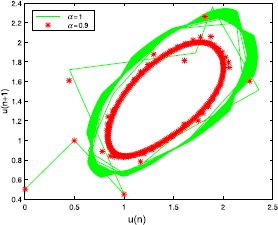
**The numerical solution of Eq. (** **3.3** **) with hybrid control NSFD scheme corresponding to** $\pmb{\tau=0.8}$ **,** $\pmb{h=1/2}$ **for different** ***α*** **.**

**Figure 5:**
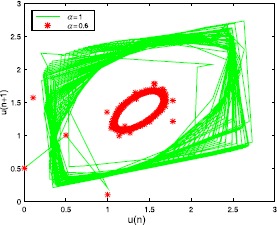
**The numerical solution of Eq. (** **3.3** **) with hybrid control NSFD scheme corresponding to** $\pmb{\tau=1.6}$ **,** $\pmb{h=1/2}$ **for different** ***α*** **.**

Let $a=30$, $b=2$, $c=2$, then $u_{\ast}=1.354$. From Table 1 we can see the different values of $\tau_{0}$ by choosing *α* with hybrid control NSFD scheme. For $h=1/2$, at different *α* and *τ*, the results refer to Figures 1-3. At the same *τ*, different *α*, the results are shown in Figures 4 and 5 (when $\alpha=1$, we obtain the results of the uncontrolled system).

**Table 1:** **The values of** $\pmb{\tau_{0}}$ **of Hybrid control NSFD scheme**

***α*** ** = 1**	$\boldsymbol {\tau_{0}}$	***α*** ** = 0.9**	$\boldsymbol {\tau_{0}}$	***α*** ** = 0.6**	$\boldsymbol {\tau_{0}}$
*h* = 1	0.4403	*h* = 1	0.5162	*h* = 1	1.0832
*h* = 1/2	0.5891	*h* = 1/2	0.6938	*h* = 1/2	1.5058

From the point of view of control, the controlled system can delay the onset of an inherent bifurcation when such a bifurcation is desired (undesired). At the same time, the parameter *α* could decide the amplitude of the closed invariant curve (Figures 4 and 5).

In fact, similar to the analysis for the NSFD control scheme, applying the Euler control method for sufficiently small step-size, we can prove the result. Through the Euler control method to Eq. (4.1), it yields the difference equation

6.1 $$ \tilde{v}_{n+1}=\tilde{v}_{n}+\alpha\tau h \bigl[a(\tilde{v}_{n-m}+u_{\ast })e^{-b(\tilde{v}_{n-m}+u_{\ast})}-c( \tilde{v}_{n}+u_{\ast}) \bigr]+(1-\alpha )\tau h \tilde{v}_{n-m}. $$

**Figure 6:**
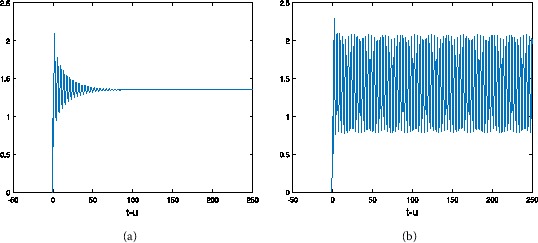
**The numerical solution of Eq. (** **3.3** **) with hybrid control Euler method corresponding to** $\pmb{\alpha=1}$ **,** $\pmb{h=1/2}$ **when (a)** $\pmb{\tau=0.4}$ **, (b)** $\pmb{\tau=0.5}$ **.**

**Figure 7:**
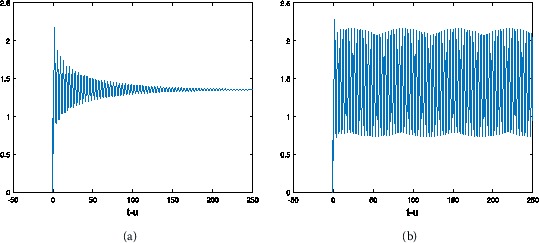
**The numerical solution of Eq. (** **3.3** **) with hybrid control Euler method corresponding to** $\pmb{\alpha=0.9}$ **,** $\pmb{h=1/2}$ **when (a)** $\pmb{\tau=0.5}$ **, (b)** $\pmb{\tau=0.6}$ **.**

**Figure 8:**
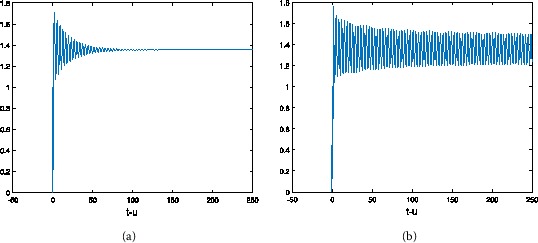
**The numerical solution of Eq. (** **3.3** **) with hybrid control Euler method corresponding to** $\pmb{\alpha=0.6}$ **,** $\pmb{h=1/2}$ **when (a)** $\pmb{\tau=0.9}$ **, (b)** $\pmb{\tau=1}$ **.**

From Figures 1-3 and Figures 6-8, Tables 1 and 2, we could argue that NSFD is better than the Euler method under the means of describing approximately the dynamics of the system with the same step-size.

**Table 2:** **The values of** $\pmb{\tau_{0}}$ **of Hybrid control Euler method**

***α*** ** = 1**	$\boldsymbol {\tau_{0}}$	***α*** ** = 0.9**	$\boldsymbol {\tau_{0}}$	***α*** ** = 0.6**	$\boldsymbol {\tau_{0}}$
*h* = 1	0.2927	*h* = 1	0.3362	*h* = 1	0.6062
*h* = 1/2	0.4452	*h* = 1/2	0.5160	*h* = 1/2	0.9914

Through the above analysis, we can improve the stability and enlarge the stable region by choosing control parameter, and thereby delay the onset of Neimark-Sacker bifurcation.

## Conclusions

In this paper, we have developed a hybrid control nonstandard finite-difference (NSFD) scheme by combining state feedback and parameter perturbation for controlling the Neimark-Sacker bifurcation in a discrete nonlinear dynamical system. In Section 3, by applying hybrid control Nicholson’s blowflies equation with delay, we obtain the Hopf bifurcation. In Section 4, compared with the results in Section 3, for any step-size, the hybrid control numerical strategy can delay the onset of an inherent bifurcation when such a bifurcation is undesired (desired) by choosing an appropriate control parameter *α*. For any step-size, we obtain the consistent dynamical results of the corresponding continuous-time model. In Section 6, numerical examples are provided to illustrate the theoretical results. Applying the Euler control method for sufficiently small step-size, we can also prove the result. We obtain that the NSFD control scheme is better than the Euler control method. There are lots of good prospects in bifurcation and control area. In the future, we can further design a better controller to control the bifurcation of Nicholson’s blowflies equation with delay.
